# The effect of 8-week Tai Chi training on emotional regulation in female college students: an ERP study of N2 and P3 under a modified oddball paradigm

**DOI:** 10.3389/fpsyg.2025.1620704

**Published:** 2025-11-06

**Authors:** Jin Yuan, Quanwen Zeng, Dan Feng, Yu Wang, Huimin Li, Zhengzhou Cong, Jiamin Xu, Anjie Wang, Jun Li, Yong Zhang

**Affiliations:** 1 School of Physical Education, Anhui Polytechnic University, Wuhu, Anhui, China; 2 School of Foreign Studies, Anhui Polytechnic University, Wuhu, Anhui, China; 3 Collaborative Innovation Center for Sports and Health Industry, Anhui Polytechnic University, Wuhu, Anhui, China

**Keywords:** Tai Chi, emotion regulation, event-related potential (ERP), aerobic exercise, cognitive control

## Abstract

**Background:**

Emotion regulation is vital for psychological well-being. Exercise can enhance regulation via attentional and cognitive control, with event-related potentials (ERPs) offering neural insights. Tai Chi benefits mood and stress, yet its neurophysiological effects remain unclear. This study examined the impact of 8-week Tai Chi training on N2 and P3 components of emotional processing in female college students using a modified oddball paradigm.

**Method:**

Forty healthy female college students were randomly assigned to an 8-week Tai Chi group (*n* = 20, 20.25 ± 1.33 yrs.) or control group (*n* = 20, 19.65 ± 1.09 yrs.). Tai Chi participants completed thrice-weekly 50 min sessions at 60–69% HRmax, including warm-up, practice, and relaxation, while controls observed without exercising. Pre- and post-intervention, all completed a modified oddball task recording N2/P3 ERPs and reaction times, alongside emotional regulation assessments, enabling comparison of neurophysiological and behavioral responses across negative, neutral, and positive stimuli.

**Results:**

After 8 weeks, the Tai Chi group exhibited significantly shorter reaction times compared to the control group (*p* < 0.001), with improvements evident only in the exercise group over time. ERP analyses revealed that Tai Chi training selectively reduced N2 amplitudes to negative stimuli, suggesting decreased early sensitivity to negative information. Additionally, the Tai Chi group showed overall shorter N2 latencies compared to the control group, indicating faster early-stage neural processing. In contrast, P3 amplitudes increased across all valence conditions in the exercise group, reflecting enhanced allocation of attentional resources during later cognitive processing. No significant effects were observed for P3 latency.

**Conclusion:**

This study demonstrates that 8 weeks of Tai Chi training modulated both neural and behavioral responses to emotional stimuli in healthy female college students, suggesting potential benefits for attentional processes in emotional regulation, though neural-behavioral links require further investigation.

## 1 Introduction

Emotion regulation refers to the processes by which individuals influence the occurrence, experience, and expression of emotions (Gross, 1998). It is essential for psychological well-being and social adaptation, playing a key role in interpersonal functioning. Exercise has been identified as an effective strategy for enhancing emotion regulation (Thayer et al., 1994; Bernstein and McNally, 2017; Edwards et al., 2017), with evidence suggesting that it optimizes attention allocation and cognitive control, thereby improving the efficiency of emotional information processing (Gomez-Pinilla and Hillman, 2013). Event-related potentials (ERPs), given their high temporal resolution, provide a powerful approach for investigating the neural mechanisms underlying these effects. Previous studies have shown that acute and chronic aerobic exercise can modulate ERP responses to emotional stimuli, including reductions in early components such as N1 and N2, suggesting reduced neural sensitivity to negative information and improved emotional stability (Zhang et al., 2022; Hwang et al., 2019).

Accumulating evidence indicates that aerobic exercise exerts both immediate and long-term benefits on emotional regulation (Long et al., 2021; Mizzi et al., 2022; Kim et al., 2022; Thom et al., 2019; Ligeza et al., 2023). Acute exercise may temporarily enhance emotion regulation through dopaminergic mechanisms that adjust arousal levels (Zheng and Hasegawa, 2016). In contrast, long-term exercise fosters more sustained adaptations, including enhanced reappraisal ability and neuroplastic changes, such as strengthened hippocampal-amygdala connectivity, improved prefrontal-amygdala white matter integrity, and increased prefrontal dopamine availability (Mizzi et al., 2022; Schmitt et al., 2020; Schaeffer et al., 2014; Chen et al., 2017). These findings highlight that regular exercise can influence both behavioral and neurophysiological markers of emotion regulation.

Tai Chi, a traditional Chinese mind–body exercise integrating movement, breath control, and focused attention, has been shown to improve mood and reduce anxiety, depression, and stress (Frith et al., 2011; Wang et al., 2004; Zheng et al., 2018; Zhang et al., 2019; Xu et al., 2021). However, most studies rely on self-report measures, with limited attention to its neural mechanisms. ERPs offer an opportunity to address this gap by examining key components involved in emotional processing. Specifically, N2, typically observed over frontal-central regions, reflects conflict monitoring, cognitive control, and early attention allocation (Carretié et al., 2004; Palomba et al., 1997). while P3, maximal over parietal regions, indexes later-stage emotional evaluation and resource allocation (Huang and Luo, 2006; Patel and Azzam, 2005; Hillman et al., 2009). Changes in these components have been linked to individual differences in emotion regulation (Lewis et al., 2006; Gardener et al., 2013), yet it remains unclear whether long-term Tai Chi training modulates these ERP markers.

The present study focuses on healthy female college students for two reasons. First, women demonstrate greater sensitivity to negative emotions than men and are more prone to emotion regulation difficulties (Bradley et al., 2001; Güntekin et al., 2017; Gard and Kring, 2007). Second, college students are at a critical developmental stage, with female students particularly vulnerable to stress and anxiety, making them an important population for intervention research. To minimize gender-related variability in ERP responses and enhance interpretability, only female participants were included. Building on evidence that regular aerobic exercise exerts measurable effects on emotion regulation after approximately 8 weeks (Mizzi et al., 2022; Zhang et al., 2019; Olson et al., 2017), and that Tai Chi has been shown to enhance neuroplasticity in college populations (Cui et al., 2019). Therefore, this study employs 8 weeks of moderate-intensity Tai Chi training to explore its neurophysiological mechanisms in emotion regulation for healthy female college students.

Using a modified oddball paradigm with negative, neutral, and positive emotional images, this study examined the effects of an 8-week Tai Chi training program on ERP and behavioral indices of emotional regulation. We hypothesized that Tai Chi would modulate the N2 and P3 components during emotional processing, reflecting potential changes in attentional and cognitive control mechanisms.

## 2 Participants and methods

### 2.1 Participants

Based on our research questions and prior knowledge in the field (Henz and Schöllhorn, 2018; Li et al., 2022; Wang et al., 2024), the sample size calculation, test types, and effect size selection were determined accordingly. Using the G*Power 3.1 program for sample size estimation, the significance level was set at *α* = 0.05, the effect size at d = 0.8, and statistical power at 1 − *β* = 0.8. *A priori* power analysis indicated that a minimum of 20 participants per group was required to achieve adequate statistical power. To account for potential attrition and data loss, 50 participants were initially recruited. Ultimately, 40 participants were included in the final analysis. Of the excluded participants, five did not meet the minimum attendance requirement, three voluntarily withdrew during the intervention period, and two were excluded due to excessive electroencephalography (EEG) artifacts that rendered their data unusable. The study participants were female students enrolled in public physical education courses at Anhui Polytechnic University, none of whom were majoring in physical education. All participants read and signed an informed consent form and received ethical approval from the Ethics Committee of the Institute of Neuroscience and Cognitive Psychology at Anhui Polytechnic University (AHPU-PED-2022-001). All procedures involving human participants were conducted in accordance with the relevant guidelines and regulations, including the Declaration of Helsinki.

Participants were screened to meet the following inclusion criteria: (1) right-handed and no reported color blindness; (2) no history of psychiatric disorders, systemic neuromuscular diseases, cardiovascular diseases, head injuries, or medications that might affect cognition; (3) no involvement in any systematic or regular physical exercise other than normal physical education activities; and (4) no experience in any school sports team within the past 3 years. In addition, none of the participants had prior experience with Tai Chi training. All participants completed the State–Trait Anxiety Inventory (STAI) (Spielberger, 1970) and the Beck Depression Inventory (BDI-II) (Beck et al., 1996) to assess their baseline psychological status, ensuring the inclusion of healthy individuals.

### 2.2 Procedures

Before the formal exercise intervention, all participants completed an informed consent form and a basic information questionnaire (e.g., medical history, age, height, etc.). The consent form detailed the organization, experiment location, experiment duration, experimental procedures, participant requirements, related rewards, and contact information. Participants could only formally join the experiment after fully understanding the research details and signing the consent form.

All participants were randomly assigned to the Tai Chi exercise group or the no-intervention control group. Based on prior instructional surveys, during the exercise intervention for the Tai Chi group, complex technical movements and movements with similar training functions were excluded. The intervention focused on easy-to-learn, easy-to-practice, and effective movements from the 24-form Tai Chi, including typical movements such as Wild Horse’s Mane, Hand Strums the Lute, Lop Knee Stance, Reverse Elbow, Grasping the Sparrow’s Tail, and Golden Rooster Stands on One Leg. This ensured that the participants could smoothly connect all technical movements during their training. The training followed a progressive approach: initially, the focus was on technique instruction to cultivate interest and help participants master the basic movements; in the later stages, technical guidance was provided to help participants establish a long-term exercise habit.

The experimental procedure consisted of three phases: During the pre-test phase, conducted within 1 week prior to the start of the intervention, all participants entered the laboratory to undergo EEG testing and psychological assessments, completing the STAI and BDI-II to evaluate baseline anxiety and depression levels. The purpose of administering these questionnaires was to confirm that all participants met the study’s inclusion criteria of being emotionally healthy, and they were therefore used for baseline screening only. The intervention lasted for 8 weeks, with participants engaging in three Tai Chi sessions per week, each lasting 50 min and consisting of 10 min of warm-up, 35 min of Tai Chi practice, and 5 min of cool-down stretching. Heart rate was continuously monitored during all training sessions using a chest strap device (Magene, China) with ECG-based detection, ensuring uninterrupted measurement. Exercise intensity was maintained at 60–69% of HRmax (120–145 beats/min), verified through real-time device feedback and cross-checked with the Rating of Perceived Exertion (RPE). To promote adherence, participants attended one group session per week, while the remaining two sessions were individually scheduled. For each individual session, participants first signed in on-site and then immediately completed the training under researcher supervision. Training logs were kept for all sessions, and adherence was verified weekly by reviewing logs and check-in records. The control group did not perform Tai Chi but followed the same schedule of three weekly sessions (one group and two individual), during which they completed the same check-in procedures and remained seated at the training site to observe, thereby matching the experimental group’s exposure to the training environment and researcher contact.

During the post-test phase, conducted within 1 week after completion of the 8-week intervention, all participants returned to the laboratory to undergo the same tests as in the pre-test phase, including EEG recordings and psychological assessments, to evaluate the effects of the exercise intervention. The control group maintained their usual daily lifestyle without any additional physical exercise throughout the experiment. During each training session, control group members remained seated at the training site, did not participate in any physical exercises, and only observed the sessions.

### 2.3 Stimuli and task

The experiment employed a modified oddball paradigm, in which each block consisted of 100 trials (70 frequent stimuli and 30 infrequent stimuli), and participants were required to respond to every stimulus using a two-choice categorization task. The standard stimulus was an image of a vase, while the deviant stimuli consisted of 10 negative, 10 neutral, and 10 positive pictures selected from the Chinese Affective Picture System (CAPS) (Lu et al., 2005). Negative images had valence ratings (M = 2.64, SD = 0.15, range = 2.37 ~ 2.88) and arousal ratings (M = 4.94, SD = 0.20, range = 4.68 ~ 5.25). Neutral images had valence ratings (M = 5.40, SD = 0.48, range = 4.63 ~ 6.04) and arousal ratings (M = 4.76, SD = 0.56, range = 4.00 ~ 6.04). Positive images had valence ratings (M = 7.25, SD = 0.11, range = 7.11 ~ 7.46) and arousal ratings (M = 6.33, SD = 0.36, range = 5.62 ~ 6.99). Ratings were assessed on a 9-point scale, with 1 indicating the least pleasant/lowest arousal and 9 indicating the most pleasant/highest arousal. Stimuli were chosen to represent prototypical examples of negative, neutral, and positive categories, with non-overlapping valence distributions to ensure clear emotional differentiation across conditions. To minimize order effects, the presentation order within each block was randomized, with standard stimuli presented at a rate of 70% and deviant stimuli at 30%. A 2 min break was provided between blocks.

**Figure 1:**
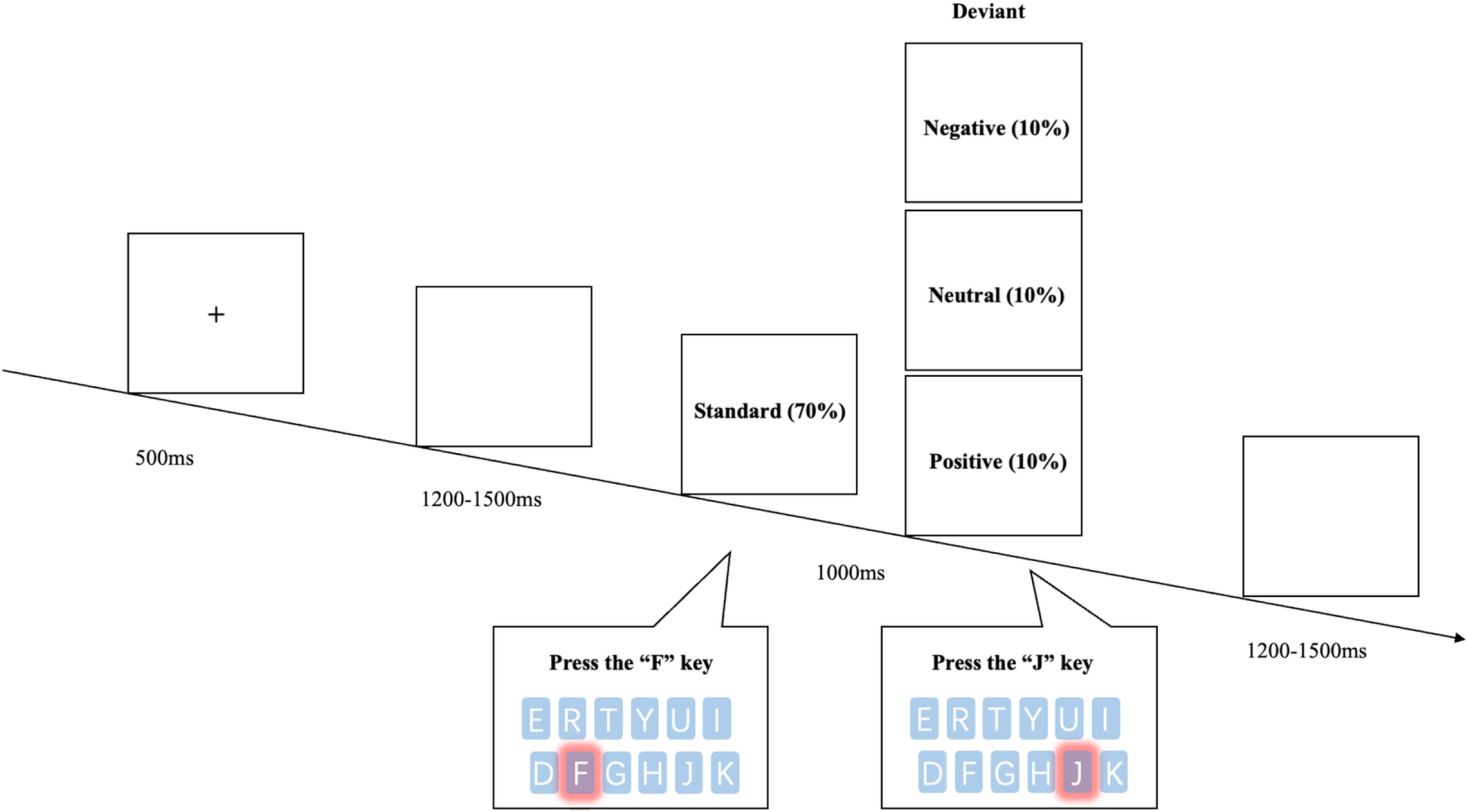
Schematic of the experimental procedure and stimuli.

The experiment was programmed using E-prime version 2.0 (Psychology Software Tools Inc., Pittsburgh, PA). The experimental task consisted of two parts: practice and formal experiment. The practice session included 10 trials, while the formal experiment consisted of 3 blocks, each containing 100 trials. Each trial began with a fixation mark “+” displayed on the computer screen for 500 ms (Figure 1). This was followed by a blank screen for 1,200 ~ 1,500 ms, after which the stimulus image appeared. The stimulus image was displayed for 1,000 ms, and participants were required to respond within this time. When a standard image appeared, participants were instructed to press the “F” key on the keyboard as quickly as possible, and when a deviant image appeared, they were instructed to press the “J” key. Reaction times (RTs) were recorded only for responses to deviant stimuli, as they were the primary focus of emotional processing in this study. Unlike the traditional oddball paradigm, in which participants typically respond only to deviant stimuli, the modified oddball paradigm adopted here required responses to both standard and deviant stimuli. This design helped sustain continuous attentional engagement, reduced response bias. Throughout the experiment, participants remained in a quiet, comfortable environment, with the computer monitor placed 70 cm from the participant.

### 2.4 ERP recording and analysis

EEG data were acquired from a non-invasive, 32-channel, gel-based mBrainTrain Smarting Pro (mBrainTrain LLC., Belgrade, Serbia). According to the international 10/20 system, the recording sites were Fp1, Fp2, F3, F4, C3, C4, P3, P4, O1, O2, F7, F8, T7, T8, TP9, P7, P8, Fz, Cz, Pz, POz, FC1, FC2, CP1, CP2, FC5, FC6, CP5, CP6, TP10, FT9, and FT10. The TP9 and TP10 electrodes were placed on the left and right mastoids, respectively, and their signals were used as offline references. Horizontal and vertical electrooculogram (EOG) activity was recorded from electrodes placed on both eyes. EEG and EOG activity were recorded with a sampling rate of 1,000 Hz using an online amplifier, and all impedance levels were kept below 10kΩ.

Offline analysis of the EEG data was performed using the open-source EEGLAB (2024.2) toolbox in MATLAB (R2021a, MathWorks Inc., United States) (Delorme and Makeig, 2004) and ERPLAB (12.00) (Lopez-Calderon and Luck, 2014). During offline analysis, EEG data were down sampled to a 500 Hz sampling rate and band-pass filtered using a finite impulse response (FIR) filter with a low-pass frequency of 30 Hz and a high-pass frequency of 0.1 Hz. The data were manually checked and artifacts related to muscle activity and eye movements were rejected. The specific steps for artefact correction and rejection were as follows: First, independent component analysis (ICA, binICA; EEGLAB) was applied to the continuous EEG data, including the EOG channels Fp1 and Fp2. Then, independent components (ICs) were classified using ICLabel (EEGLAB plugin) to identify potential artefact components. If a component had more than 80% eye movement-related activity and less than 10% brain-related activity, it was marked as a potential artefact and excluded (Sanada and Katayama, 2024). For each experimental condition, EEG activity was averaged independently, and baseline correction and feature extraction were performed within the time window of 200 ms before to 1,000 ms after stimulus presentation. EEG data were then time-locked to the onset of deviant stimuli, and epochs belonging to the same experimental condition were averaged to generate the average ERP waveform for each participant under the Negative, Neutral, and Positive stimulus conditions. Consistent with previous ERP studies (Hwang et al., 2019; Qiu et al., 2019; Yuan et al., 2008; Moser et al., 2006; Buodo et al., 2017), visual inspection of the grand-averaged ERP waveforms elicited by the stimuli indicated that the N2 component emerged in the 210–290 ms time window at frontal-central scalp sites (i.e., Fz, Cz, FC1, and FC2 electrodes), and the P3 component emerged in the 300–400 ms time window at parietal scalp sites (i.e., P3, Pz, and P4 electrodes). To illustrate the spatial distributions of these components, scalp topographies of N2 (210–290ms) and P3 (300–400ms) at post-test for the Tai Chi and control groups under negative, neutral, and positive emotional conditions are provided in Supplementary Figure 1. For statistical analysis, Fz and Pz were chosen as representative sites, as they showed the most pronounced amplitudes of N2 and P3, respectively.

### 2.5 Statistical analyses

All statistical analyses were conducted in IBM SPSS version 27 (IBM Corp., Armonk, NY). RTs were averaged across trials for each emotional condition (negative, neutral, and positive) and participant. Group differences at baseline were first assessed using independent-samples *t*-tests. Subsequently, a 2 (Group: exercise vs. non-exercise) × 2 (Time: pre- vs. post-intervention) × 3 (Emotional Valence: negative vs. neutral vs. positive) mixed-design repeated-measures ANOVA (RM-ANOVA) was performed on mean RTs, with Group as the between-subjects factor and Time and Emotional Valence as within-subjects factors. For each participant, mean amplitude and peak latency were extracted for N2 (210–290ms, Fz electrode) and P3 (300–400ms, Pz electrode). The same 2 × 2 × 3 RM-ANOVA design was applied separately for N2 and P3 mean amplitude and latency. Significant main effects and interactions were followed up with Bonferroni-corrected pairwise comparisons. For significant interactions, simple main effects analyses were conducted. When sphericity assumptions were violated, Greenhouse–Geisser corrections were applied (Jennings and Wood, 1976). Effect sizes were reported as partial eta squared (ηp^2^), and significance was set at *p* < 0.05.

## 3 Results

As shown in Table 1, baseline characteristics measured during the pre-test were compared between the exercise and non-exercise groups. Independent samples *t*-tests revealed no significant differences between the exercise group and the non-exercise group in age, BMI, STAI, BDI-II, or reaction times to the three types of deviant stimuli (*p* > 0.05).

**Table 1:** Baseline characteristics of the exercise and non-exercise groups.

Variable	Exercise	Non-exercise	Statistics	*p*
Mean age (years)	20.25 ± 1.33	19.65 ± 1.09	*t*(38) = 1.56	0.127
Mean BMI	21.82 ± 1.82	21.02 ± 1.93	*t*(38) = 1.34	0.187
Mean STAI anxiety score	42.00 ± 5.13	41.15 ± 5.09	*t*(38) = 0.526	0.602
Mean BDI-II depressiveness score	8.15 ± 2.92	8.25 ± 2.31	*t*(38) = 0.12	0.905
RT for Negative deviants (ms)	492.82 ± 76.47	508.12 ± 59.56	*t*(38) = 0.706	0.485
RT for Neutral deviants (ms)	510.46 ± 61.68	516.93 ± 61.79	*t*(38) = 0.331	0.742
RT for Positive deviants (ms)	529.29 ± 75.23	515.69 ± 89.28	*t*(38) = 0.521	0.605

M ± SD.

### 3.1 Reaction times

**Figure 2:**
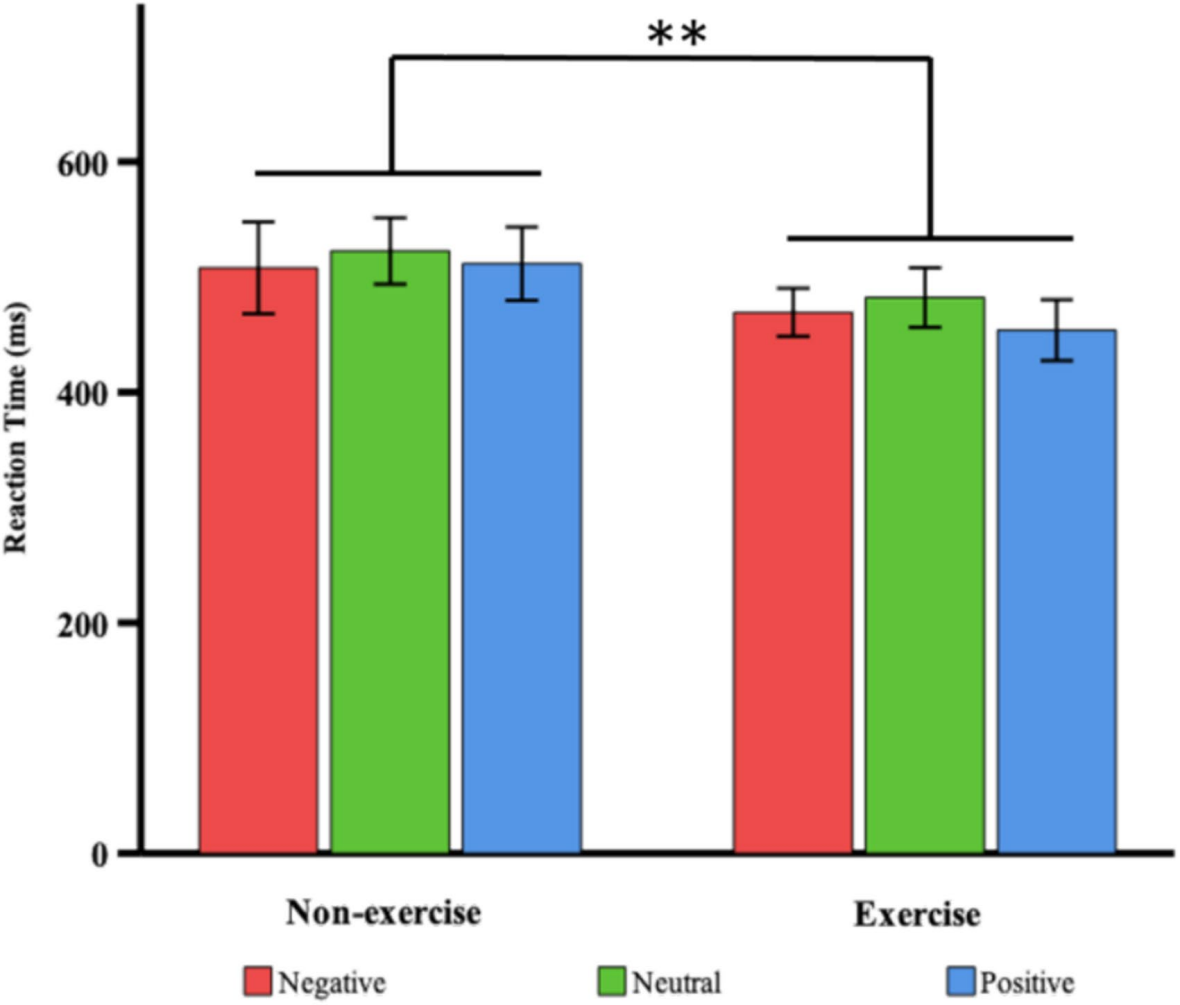
Comparisons of RTs in response to deviant stimuli, by valence type, between the exercise and non-exercise groups. Relative to the non-exercise group, the exercise group had faster RTs for negative (red), neutral (green) and positive (blue) deviant stimuli. Means are shown with standard errors; ** *p* < 0.01.

A two-way (valence × group) ANOVA on the RTs to deviant emotional stimuli revealed a significant main effect of group after 8 weeks, *F*(1, 38) = 18.761, *p* < 0.001, η^2^ = 0.331, indicating that the exercise group of female college students exhibited significantly shorter RTs compared to the non-exercise group (468.51 ± 27.20 ms vs. 514.06 ± 38.36 ms). The main effect of valence was not significant, *F*(2, 76) = 0.949, *p* = 0.392. The interaction effect between valence and group was also not significant, *F*(2, 76) = 0.26, *p* = 0.772 (see Table 2; Figure 2). Therefore, regardless of the valence of the stimulus, female college students in the exercise group responded more quickly to deviant stimuli than those in the non-exercise group.

**Table 2:** Comparison of emotional valence, reaction times, amplitudes, and latencies across exercise and non-exercise groups.

Valence	Non-Exercise group (*n* = 20)						Exercise group (*n* = 20)					
	Pre-test			Post-test			Pre-test			Post-test		
	Negative	Neutral	Positive	Negative	Neutral	Positive	Negative	Neutral	Positive	Negative	Neutral	Positive
RT	508.12 ± 59.56	516.93 ± 61.79	515.69 ± 89.28	507.91 ± 84.96	522.65 ± 61.49	511.62 ± 67.96	492.82 ± 76.47	510.46 ± 61.68	529.29 ± 75.23	469.38 ± 44.53^a^	482.25 ± 55.50^a^^,^^b^	453.91 ± 56.37^a^^,^^b^
MRT	513.58 ± 40.95			514.06 ± 38.36			510.86 ± 41.66			468.51 ± 27.20^a^^,^^b^		
Amplitude (μV)												
N2 (−)	11.28 ± 4.14	7.56 ± 5.29	7.79 ± 5.46	11.52 ± 5.70	7.78 ± 5.31	7.95 ± 5.59	11.59 ± 3.99	8.36 ± 4.66	7.92 ± 4.74	8.38 ± 2.89^a^^,^^b^	8.04 ± 5.86	8.31 ± 6.45
P3 (+)	6.78 ± 3.55	5.91 ± 2.82	6.15 ± 4.05	6.88 ± 4.43	6.10 ± 3.63	6.39 ± 4.32	6.46 ± 4.12	5.67 ± 3.60	6.49 ± 4.62	9.93 ± 4.07^a^^,^^b^	8.95 ± 3.73^a^^,^^b^	9.24 ± 4.66
Latency (ms)												
N2	256.50 ± 24.59	262.10 ± 28.40	265.00 ± 23.46	254.90 ± 21.11	255.30 ± 19.68	253.60 ± 25.79	249.70 ± 30.86	245.50 ± 26.42	241.60 ± 32.72	253.30 ± 18.50	253.20 ± 20.64	246.30 ± 21.50
P3	352.60 ± 32.79	347.60 ± 24.03	355.30 ± 28.93	364.60 ± 25.70	360.40 ± 31.38	365.60 ± 20.75	357.30 ± 35.16	351.00 ± 29.66	354.60 ± 35.14	368.40 ± 27.94	358.70 ± 28.59	356.80 ± 30.61

Data are presented as the mean ± SD. RT represents reaction time; MRT represents mean reaction time.

a Significant difference between post-test and pre-test, *p* < 0.05.

b Significant difference between non-exercise group and exercise group, *p* < 0.05.

**Figure 3:**
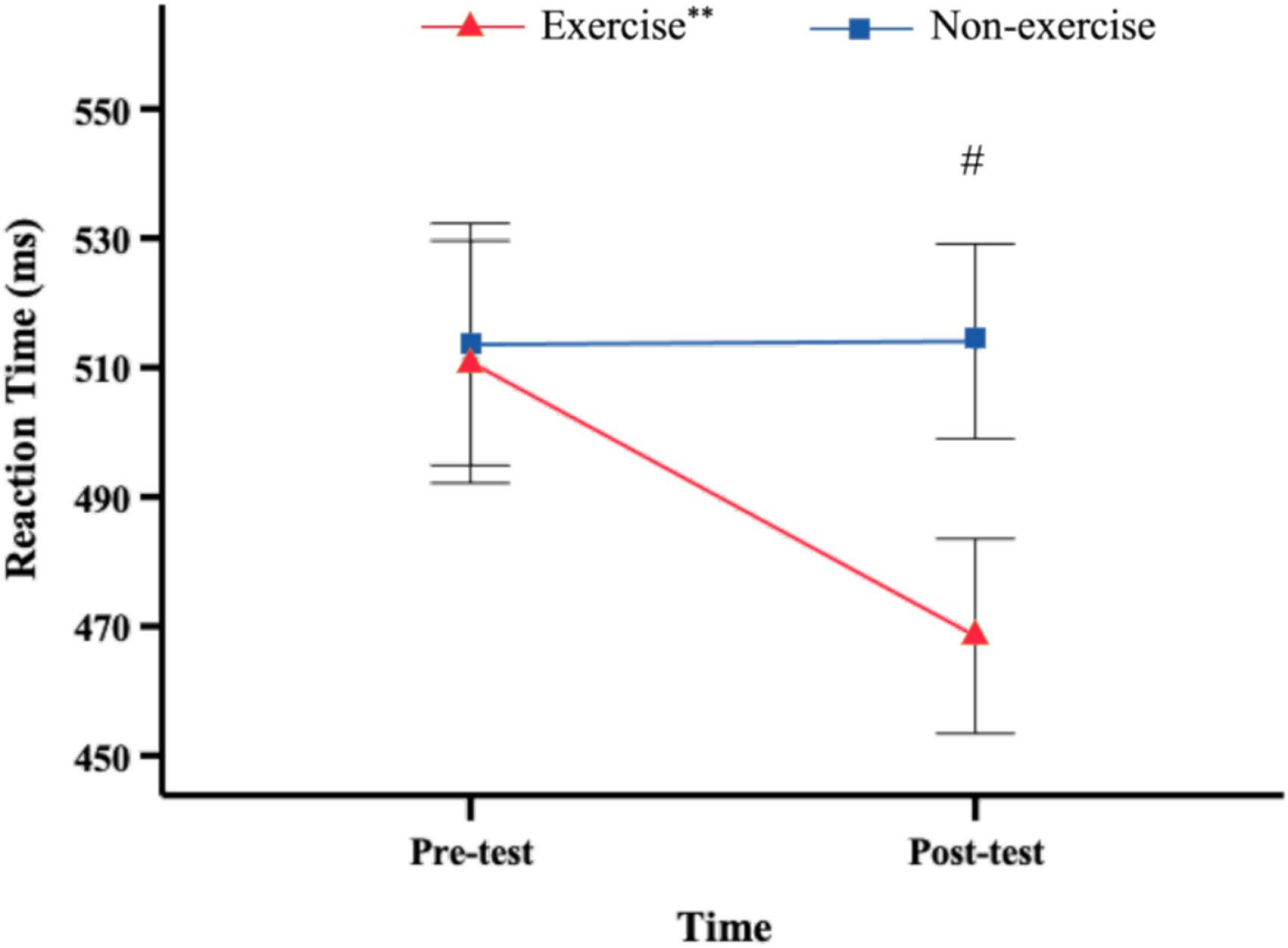
Reaction times (mean and SEM) as a function of time point. ** Significant difference between pre-test and post-test for this group, *p* < 0.01. # Significant difference between groups, *p* < 0.05.

The results on reaction time revealed a significant main effect of time, *F*(1, 38) = 9.628, *p* = 0.004, η^2^ = 0.202. The main effect of group was also significant, *F*(1, 38) = 6.126, *p* = 0.018, η^2^ = 0.139. The interaction effect between time and group was significant as well, *F*(1, 38) = 10.078, *p* = 0.003, η^2^ = 0.21. Further simple effect analysis indicated that there was no significant change in RTs between pre-test and post-test measurements for the non-exercise group, *F*(1, 38) = 0.003, *p* = 0.96. However, the exercise group exhibited significantly lower RTs at the post-test compared to the pre-test, *F*(1, 38) = 19.704, *p* < 0.001, η^2^ = 0.341 (Figure 3).

### 3.2 ERP results

**Figure 4:**
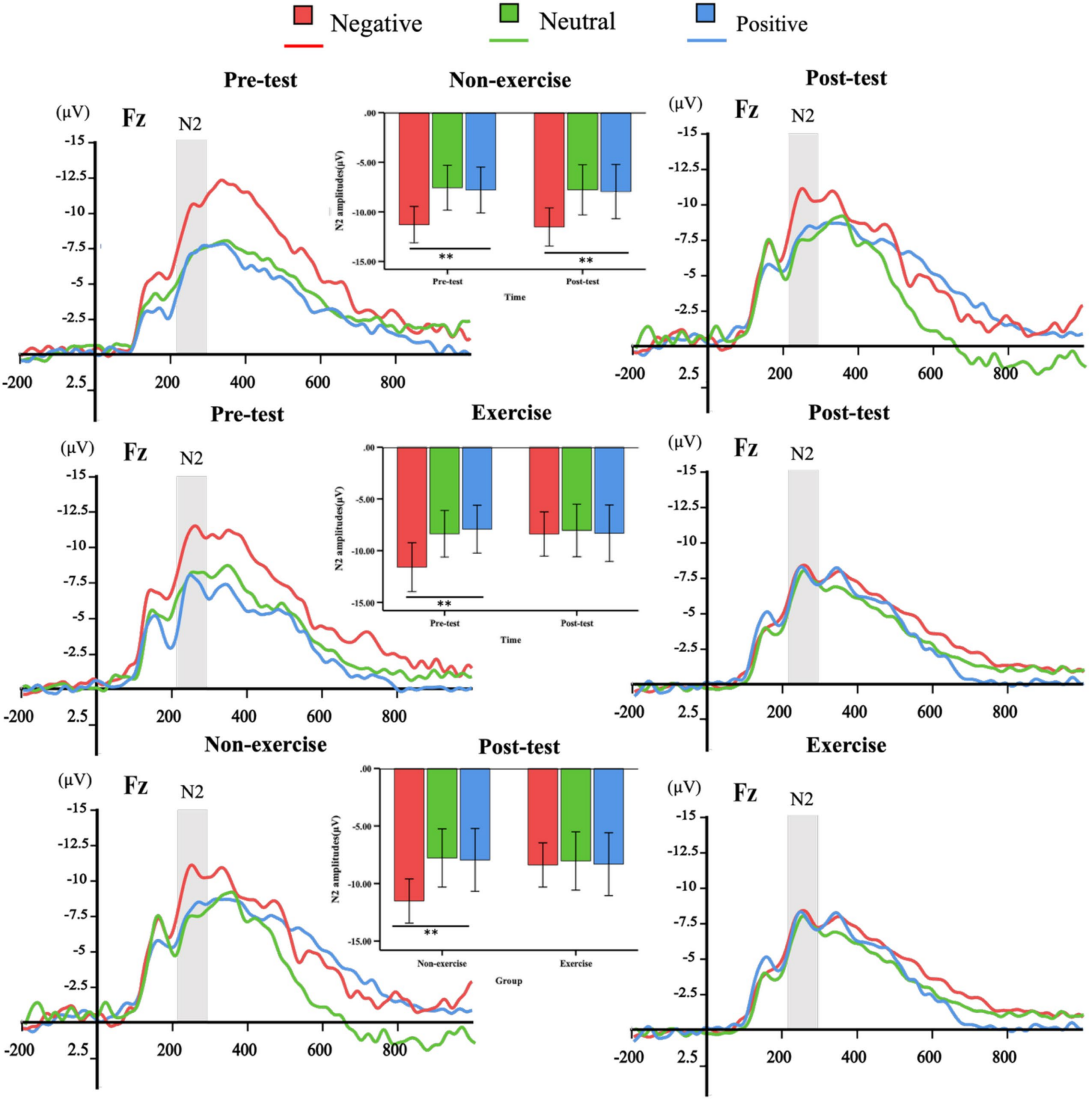
The overall averages of ERPs induced by deviant stimuli in the exercise and non-exercise groups. ERP waveforms triggered by negative (red), neutral (green), and positive (blue) deviant stimuli were measured at the representative frontal electrode (Fz). Additionally, N2 (210–290ms) amplitude plots for both pre-test and post-test within-group measurements, as well as between-group post-test comparisons, are presented.

**Figure 5:**
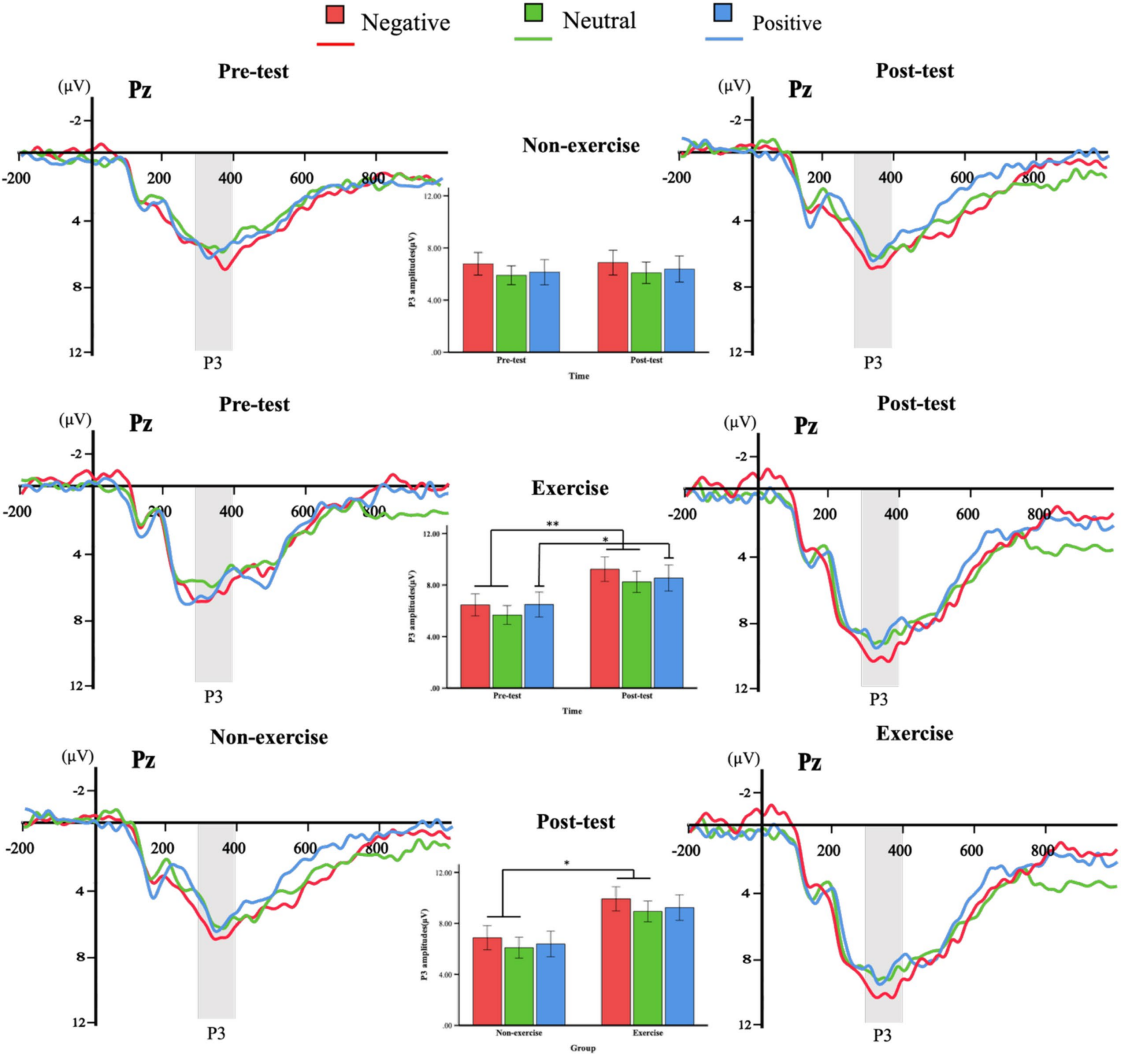
Grand averages of ERPs induced by deviant stimuli in the exercise and non-exercise groups. ERP waveforms evoked by negative (red), neutral (green), and positive (blue) deviant stimuli were measured at the representative parietal electrode (Pz), along with the P3 (300–400ms) amplitude plots for both groups at pre-test, post-test, and between-group comparisons at post-test.

The group-level ERP waveforms, along with the mean amplitudes and peak latencies of N2 and P3 responses to deviant emotional stimuli, are presented in Figures 4, 5 and summarized in Table 2. The results of the repeated-measures ANOVAs for N2 and P3 amplitude and latency are summarized in Table 3. To further support the observed spatial distribution patterns, scalp topographies of N2 (210–290ms) and P3 (300–400ms) components at post-test for both groups are provided in Supplementary Figure 1.

**Table 3:** Results of repeated-measures ANOVAs for N2 and P3 amplitude and latency.

ERP component	Effect	F(df)	*P*	η^2^	Significance
N2 amplitude	Group	*F*(1, 38) = 0.037	0.849	0.001	–
	Valence	*F*(2, 76) = 23.687	0.000	0.384	*
	Time	*F*(1, 38) = 0.192	0.664	0.005	–
	Group × valence	*F*(2, 76) = 2.620	0.101	0.065	–
	Group × time	*F*(1, 38) = 0.422	0.520	0.011	–
	Valence × time	*F*(2, 76) = 4.305	0.029	0.102	*
	Group × valence × time	*F*(2, 76) = 4.600	0.024	0.108	*
N2 latency	Group	*F*(1, 38) = 7.756	0.008	0.170	*
	Valence	*F*(2, 76) = 0.287	0.751	0.008	–
	Time	*F*(1, 38) = 0.020	0.889	0.001	–
	Group × valence	*F*(2, 76) = 1.364	0.262	0.035	–
	Group × time	*F*(1, 38) = 1.742	0.195	0.044	–
	Valence × time	*F*(2, 76) = 0.275	0.767	0.007	–
	Group × valence × time	*F*(2, 76) = 0.408	0.665	0.011	–
P3 amplitude	Group	*F*(1, 38) = 2.579	0.117	0.064	–
	Valence	*F*(2, 76) = 4.905	0.014	0.114	*
	Time	*F*(1, 38) = 4.316	0.045	0.102	*
	Group × valence	*F*(2, 76) = 0.159	0.822	0.004	–
	Group × time	*F*(1, 38) = 3.454	0.071	0.083	–
	Valence × time	*F*(2, 76) = 0.240	0.765	0.006	–
	Group × valence × time	*F*(2, 76) = 0.479	0.602	0.012	–
P3 latency	Group	*F*(1, 38) = 0.001	0.980	0.000	–
	Valence	*F*(2, 76) = 0.954	0.387	0.024	–
	Time	*F*(1, 38) = 2.350	0.133	0.058	–
	Group × valence	*F*(2, 76) = 0.492	0.605	0.013	–
	Group × time	*F*(1, 38) = 0.401	0.530	0.010	–
	Valence × time	*F*(2, 76) = 0.221	0.797	0.006	–
	Group × valence × time	*F*(2, 76) = 0.095	0.906	0.002	–

* Indicates *p* < 0.05.

#### 3.2.1 N2 amplitude and latency

A three-way (valence × group × time) repeated-measures ANOVA conducted on the N2 amplitude induced by deviant emotional stimuli revealed a significant main effect of valence, *F*(2, 76) = 23.687, *p* < 0.001, η^2^ = 0.384, indicating that the N2 amplitude induced by negative stimuli was significantly higher than that induced by neutral and positive stimuli. Furthermore, the group × time interaction effect was significant, *F*(2, 76) = 4.305, *p* = 0.029, η^2^ = 0.102, with the N2 amplitude of the exercise group being significantly lower at post-test compared to pre-test. Additionally, a significant valence × group × time interaction was observed, *F*(2, 76) = 4.60, *p* = 0.024, η^2^ = 0.108.

To explore further, simple main effects analyses were conducted. In the non-exercise group, a significant valence × time interaction effect was found (pre-test: *F*(2, 37) = 12.934, *p* < 0.001, η2 = 0.411; post-test: *F*(2, 37) = 7.437, *p* = 0.002, η^2^ = 0.287). The N2 amplitude induced by negative stimuli was significantly higher than that induced by neutral (*p* < 0.01) and positive (*p* < 0.01) stimuli in both pre-test and post-test. However, there were no significant differences between neutral and positive stimuli in either pre-test or post-test (*p* > 0.05).

In the exercise group, a significant valence × time interaction effect was also found, *F*(2, 37) = 11.095, *p* < 0.001, η^2^ = 0.375. The N2 amplitude induced by negative stimuli was significantly higher than that induced by neutral (*p* < 0.01) and positive (*p* < 0.01) stimuli in the pre-test, but no significant differences were observed among the valence conditions in the post-test (*p* > 0.05).

At the post-test, there was a significant difference in the N2 amplitude between the exercise and non-exercise groups, *F*(1, 38) = 5.426, *p* = 0.025, η^2^ = 0.125. Further analysis revealed that, under the negative stimulus condition, the N2 amplitude of the exercise group was significantly lower than that of the non-exercise group (*p* = 0.025), whereas no significant differences were observed between the two groups under neutral and positive stimulus conditions (*p* > 0.05). These results suggest that the 8-week exercise intervention primarily influenced the processing of negative emotional stimuli. The three-way repeated-measures ANOVA (valence × group × time) for N2 latency revealed a significant main effect of group (*F*(1, 38) = 7.756, *p* = 0.008, η^2^ = 0.170), indicating that the Tai Chi group exhibited overall shorter N2 latencies compared to the control group. However, no significant main effects of valence or time, and no significant interactions were observed (all *p* > 0.05).

#### 3.2.2 P3 amplitude and latency

A three-way (group × valence × time) repeated measures ANOVA analysis of P3 amplitude revealed a significant main effect of valence, *F*(2, 76) = 4.905, *p* = 0.014, η^2^ = 0.114, indicating significant differences in P3 amplitude across different emotional stimulus valence conditions. Post-hoc tests further showed that the P3 amplitude induced by negative stimuli was significantly higher than that induced by neutral stimuli (*p* = 0.02), while the differences between negative and positive stimuli, as well as neutral and positive stimuli, were not significant (*p* > 0.05). The main effect of time was also significant, *F*(1, 38) = 4.316, *p* = 0.045, η^2^ = 0.102, with post-test P3 amplitude being significantly higher than pre-test, suggesting an independent enhancement of attentional allocation over time. The main effect of group was not significant, *F*(1, 38) = 2.579, *p* = 0.117, η^2^ = 0.064. None of the interactions reached conventional significance, although the group × time interaction was marginal, *F*(1, 38) = 3.454, *p* = 0.071, η^2^ = 0.083. Although the main effect of group was non-significant, this marginal interaction justified exploratory post-hoc analyses.

Exploratory pairwise comparisons revealed no significant differences in P3 amplitude across valence conditions in the non-exercise group (*p* > 0.05). In the exercise group, post-test amplitudes were significantly higher than pre-test for negative (*p* = 0.007), neutral (*p* = 0.005), and positive stimuli (*p* = 0.036), indicating enhanced late-stage attentional processing following Tai Chi training. Between-group comparisons at post-test showed higher P3 amplitudes in the exercise group for negative (*p* = 0.029) and neutral stimuli (*p* = 0.019), while the difference for positive stimuli did not reach significance (*p* = 0.052). These between-group comparisons are considered exploratory due to the marginal group × time interaction.

The three-way repeated measures ANOVA (valence × group × time) for P300 latency under emotional stimuli showed no significant main effects (all *p* > 0.05) and no significant interaction (*F*(2, 76) = 0.095, *p* = 0.906).

## 4 Discussion

This study utilized ERP technology combined with behavioral measures to investigate the effects of 8 weeks of Tai Chi training on emotional regulation ability in healthy female college students. The results indicated that the RTs in a modified oddball task were significantly shortened in the training group, suggesting that individuals’ information processing efficiency during emotional processing tasks was enhanced. Additionally, ERP data revealed a significant decrease in the N2 amplitude under negative emotional stimuli in the training group, indicating a reduced sensitivity to negative information and a subsequent decrease in emotional conflict. For P3 amplitude, significant main effects of valence and time were observed, while the group main effect was non-significant and the group × time interaction was marginal, indicating overall enhancement of attentional allocation over time. Exploratory analyses showed that P3 amplitude increased across all valence conditions in the training group but not in the non-exercise group, suggesting that Tai Chi selectively enhances late-stage attentional processing. Given the marginal interaction, between-group differences should be interpreted cautiously. These findings highlight the beneficial effects of Tai Chi training on emotional regulation, although the observed improvements may also reflect general benefits of aerobic exercise rather than mechanisms unique to Tai Chi.

Specifically, the significantly shorter RTs observed in the training group relative to the control group suggest that Tai Chi training enhanced participants’ attentional control and cognitive flexibility, thereby facilitating faster processing of target stimuli. This finding is consistent with prior research showing that aerobic exercise can improve executive functions and increase the efficiency of cognitive information processing and visual target detection (Chen and Ringenbach, 2016; Fu and Yang, 2024; Gao et al., 2020; Wang et al., 2014; Komiyama et al., 2023). In parallel with the behavioral evidence, the Tai Chi group also exhibited significantly shorter N2 latencies compared to the control group, indicating faster neural responses during emotional stimulus processing. Shorter N2 latencies are generally interpreted as reflecting enhanced efficiency in early-stage conflict monitoring and cognitive control mechanisms (Schubert et al., 2023). This neural acceleration suggests that long-term Tai Chi practice may optimize the timing of attentional engagement, enabling participants to detect and evaluate emotional stimuli more rapidly. Taken together, the shortened RTs and N2 latencies provide converging behavioral and neural evidence that Tai Chi facilitates a more efficient coordination between early neural processing and subsequent behavioral responses during emotional regulation tasks. Importantly, these behavioral enhancements can also be interpreted through the lens of Tai Chi as an action-based mindfulness practice that cultivates a calm-energetic state. With repeated practice, participants may develop a tonic component of sustained calm readiness (Schumann et al., 2022), reducing susceptibility to distraction, alongside a phasic component that allows flexible entry into a focused, energetically engaged state when required (Steghaus and Poth, 2024). This dual mechanism provides a plausible explanation for the observed improvements in attentional control, as reflected in shorter RTs. While Tai Chi emphasizes coordinated movement, breathing control, and attentional focus, which may provide additional cognitive benefits (Li et al., 2022), the current study cannot definitively determine whether these effects are unique to Tai Chi or reflect broader exercise-related mechanisms.

Moreover, the observed reduction in N2 amplitude further underscores the beneficial effect of Tai Chi training on emotional regulation. N2, a neurophysiological indicator of emotional sensitivity (Yang et al., 2018; Li et al., 2008; Yuan et al., 2009), reflects early processing of emotional stimuli and is typically associated with cognitive control, conflict monitoring, and the allocation of attention resources (Botvinick et al., 2001; Folstein and Van Petten, 2008; Kumar et al., 2009). In this study, after 8 weeks of Tai Chi training, the training group showed a significant decrease in the average N2 amplitude under negative emotional stimuli compared to pre-training measurements. This result may be associated with reduced sensitivity to negative information, thereby lowering the vigilance and processing demands for negative stimuli during the early attention allocation phase and freeing up more resources for subsequent cognitive processes. Notably, this modulation may correspond to enhanced tonic calm readiness developed through Tai Chi practice (Schumann et al., 2022), enabling participants to reduce early conflict monitoring demands and reallocate cognitive resources more efficiently, consistent with the theoretical framework that Tai Chi promotes sustained attention and energetic engagement. Behavioral data further support this interpretation, as the training group demonstrated significantly shorter RTs in the modified oddball task, indicating more efficient emotional information processing. In line with these findings, Wang et al. (2023) demonstrated that Tai Chi practice improved individuals’ visual working memory capacity and narrowed emotional fluctuations between positive and negative stimuli, suggesting enhanced emotion regulation through attentional control mechanisms. Their results provide converging evidence that Tai Chi’s attentional demands and posture-memory coordination strengthen both cognitive capacity and emotion regulation. This perspective supports the interpretation that reduced N2 amplitudes in the present study reflect improved early attentional filtering of negative stimuli, consistent with Tai Chi’s facilitative effects on working memory and emotional stability.

When considered alongside the P3 findings, the decrease in N2 amplitude appears to facilitate more efficient late-stage attentional processing, suggesting that participants experienced reduced early interference from negative stimuli and could subsequently allocate attentional resources more effectively during target evaluation. The potential mechanism underlying the changes in N2 due to exercise may involve the modulation effect of the prefrontal-limbic system. Previous studies have shown that acute aerobic exercise can enhance the regulation of emotional processing by the dorsolateral prefrontal cortex (DLPFC) and reduce amygdala hyper-reactivity, thereby reducing excessive attention to negative stimuli (Wang et al., 2024). Over time, consistent engagement in physical training may induce steady-state adaptations and neuroplastic changes that strengthen emotion-regulatory control (Weng et al., 2016). Moreover, regular exercise can modulate the sympathetic nervous system and the hypothalamic–pituitary–adrenal (HPA) axis, attenuating physiological stress responses to negative emotions—a mechanism that may further support emotion regulation (Mikkelsen et al., 2017). Interestingly, our study found that the training group did not exhibit similar changes in N2 amplitude under neutral emotional stimuli, and no significant modulation was observed for positive emotional stimuli. This pattern aligns with previous research (Zhang et al., 2022), indicating that Tai Chi training may have exert a selective regulatory effect on negative emotional processing, with relatively limited impact on neutral or positive emotions.

The P3 component, a well-established marker of late-stage cognitive processing, reflects the allocation of attentional and cognitive resources during the evaluation of emotional stimuli (Huang and Luo, 2006; Gardener et al., 2013; Hajcak et al., 2010). In the present study, a significant main effect of valence and time was observed, while group-related effects did not reach significance. This indicates that emotional content and measurement time independently influenced P3 amplitude, with a general increase from pre- to post-test across all participants. Although the group × time interaction was only marginally significant (*p* = 0.071), exploratory analyses suggested a tendency for larger post-test P3 amplitudes in the Tai Chi training group, particularly for negative and neutral stimuli. This trend may imply enhanced attentional engagement and improved cognitive resource allocation following the intervention. Consistent with prior evidence linking larger P3 amplitudes to heightened attention and more efficient resource distribution (Donchin and Coles, 1988; Hillman et al., 2012), these findings suggest that Tai Chi may facilitate a state of adaptive attentional readiness rather than produce a statistically robust group effect. When considered alongside the shorter RTs and reduced N2 amplitudes, this pattern implies indicate that Tai Chi may optimize both early and late stages of emotional information processing by reducing initial conflict monitoring demands and enhancing attentional resource allocation during later cognitive operations.

Importantly, the observed time-dependent increase in P3 amplitude—regardless of group—indicates improved attentional efficiency over repeated testing, potentially reflecting learning or task-familiarity effects rather than intervention-specific outcomes. Nevertheless, the directional trend toward higher P3 amplitudes in the Tai Chi group aligns with literature demonstrating that regular physical activity enhances top-down attentional control and emotion regulation (Hillman et al., 2008; Kamijo and Takeda, 2010). The absence of an active exercise control group, however, limits our ability to distinguish Tai Chi-specific effects from the broader cognitive benefits of moderate-intensity aerobic exercise. Future research incorporating exercise-based control conditions and longitudinal neuroimaging approaches will be critical for delineating Tai Chi’s unique contributions to emotional and cognitive regulation.

Despite the valuable insights provided by this study on the effects of Tai Chi training on emotional regulation in female college students using ERP measures, several limitations should be acknowledged. First, the relatively small sample size (about 20 participants per group) reduces statistical power, particularly for higher-order interactions such as Group × Valence, which typically require 30–35 participants or more per group for stable and replicable estimates. Although the observed ERP and behavioral effects were significant and consistent with prior research, small samples may inflate effect sizes and limit generalizability. Second, the pre–post design over an eight-week period captures only short-term effects, leaving open the question of whether the observed benefits are sustained. Longitudinal studies with follow-up assessments are needed to determine the durability of Tai Chi’s effects and their neural underpinnings. Third, the study focused exclusively on N2 and P3 components, omitting other potentially informative electrophysiological or physiological indicators (e.g., P1, N1, HRV). Additionally, while the study included state–trait anxiety and depression measures, these instruments do not fully capture the experiential aspects of Tai Chi, such as momentary vitality, relaxation, and bodily ease. Tai Chi, as an action-based mindfulness practice, may modulate energetic and tense arousal in ways not reflected by traditional anxiety or affective scales. Future research could adopt circumplex-based state measures to better situate ERP and behavioral effects within participants’ lived experiences. Fourth, RT data for standard stimuli were not preserved, which restricts the ability to fully rule out general motor effects. Fifth, the absence of an active exercise control group limits interpretability; it remains unclear whether the observed improvements are specific to Tai Chi or reflect general benefits of moderate-intensity aerobic exercise. Future work should incorporate appropriate exercise control conditions to disentangle these effects. Moreover, despite controlling for intervention duration and intensity, potential confounding factors such as psychological states, lifestyle habits, and individual differences (e.g., sleep quality, social support) may have influenced outcomes, warranting stricter control in future studies. Finally, the study used a modified oddball paradigm (70–30 distribution) requiring responses to both frequent and infrequent stimuli. Unlike the classical oddball that captures unexpected rare events, this design reflects frequency discrimination, so results should be interpreted within this context.

## 5 Conclusion

This study demonstrated that 8 weeks of Tai Chi training modulated both neural (N2, P3) and behavioral (RT) responses to emotional stimuli in healthy female college students, suggesting potential benefits for attentional and cognitive processes in emotional regulation. However, as the study did not directly test the associations between ERP changes and behavioral outcomes, interpretations regarding more “effective” attentional allocation should be made with caution. Future research should examine these neural-behavioral links to strengthen the evidence base.

## Data Availability

The original contributions presented in the study are included in the article/Supplementary material, further inquiries can be directed to the corresponding author.
