# Supplementary material for: The effect of 8-week Tai Chi training on emotional regulation in female college students: an ERP study of N2 and P3 under a modified oddball paradigm
Source: Front Psychol. 2025 Nov 6;16:1620704. doi: 10.3389/fpsyg.2025.1620704 (PMC12631280; doi:10.3389/fpsyg.2025.1620704)
Supplement: Supplementary file 1 [file Supplementary_file_1.docx]

**Supplementary Materials**


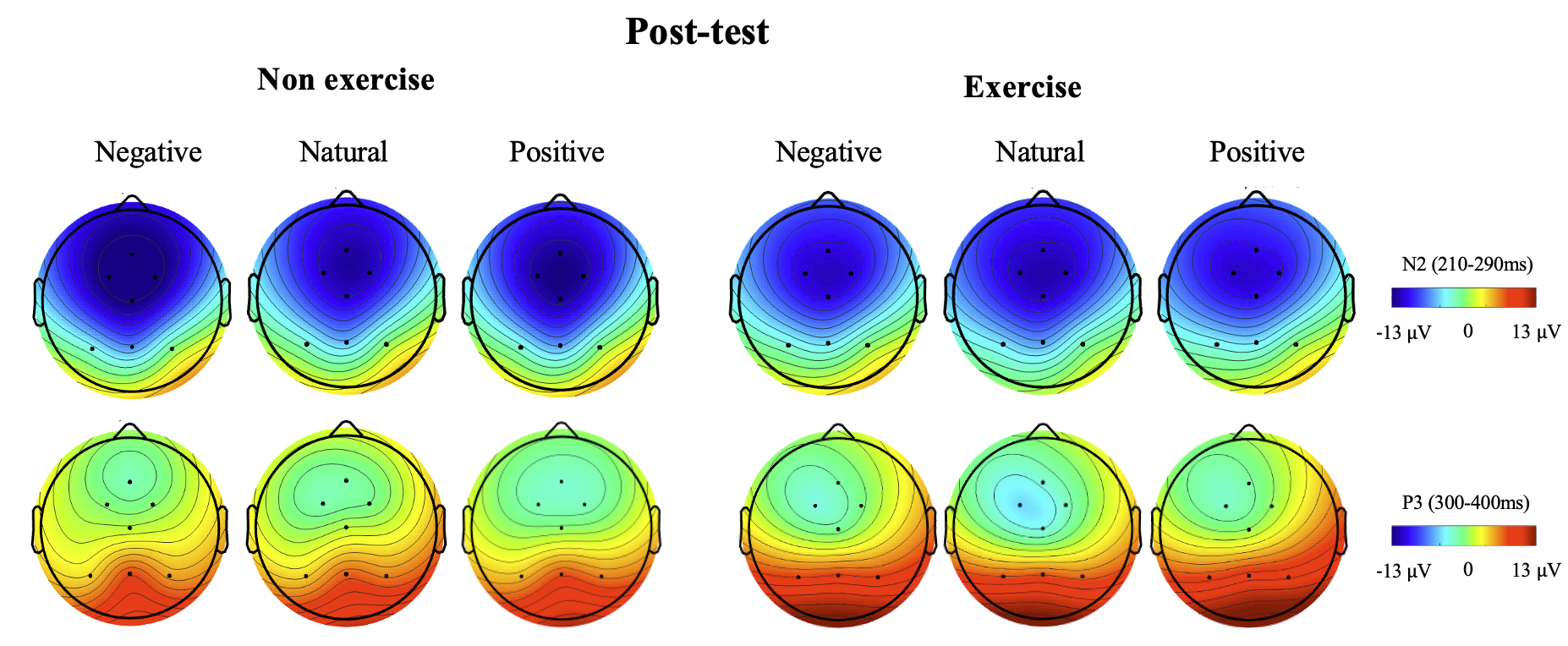


**Fig S1.** Scalp topographies of N2 (210-290ms) and P3 (300-400ms) components at post-test for the Tai Chi (exercise) and control groups under negative, neutral, and positive emotional conditions.
